# A Force-Based, Parallel Assay for the Quantification of Protein-DNA Interactions

**DOI:** 10.1371/journal.pone.0089626

**Published:** 2014-02-27

**Authors:** Katja Limmer, Diana A. Pippig, Daniela Aschenbrenner, Hermann E. Gaub

**Affiliations:** 1 Lehrstuhl für Angewandte Physik and Center for Nanoscience (CeNS), Ludwig-Maximilians-University, Munich, Germany; 2 Munich Center for Integrated Protein Science (CIPSM), Munich, Germany; Florida International University, United States of America

## Abstract

Analysis of transcription factor binding to DNA sequences is of utmost importance to understand the intricate regulatory mechanisms that underlie gene expression. Several techniques exist that quantify DNA-protein affinity, but they are either very time-consuming or suffer from possible misinterpretation due to complicated algorithms or approximations like many high-throughput techniques. We present a more direct method to quantify DNA-protein interaction in a force-based assay. In contrast to single-molecule force spectroscopy, our technique, the Molecular Force Assay (MFA), parallelizes force measurements so that it can test one or multiple proteins against several DNA sequences in a single experiment. The interaction strength is quantified by comparison to the well-defined rupture stability of different DNA duplexes. As a proof-of-principle, we measured the interaction of the zinc finger construct Zif268/NRE against six different DNA constructs. We could show the specificity of our approach and quantify the strength of the protein-DNA interaction.

## Introduction

The sequence-specific interaction of certain proteins with the genomic DNA is prerequisite for the complex task of transcriptional regulation. Those transcription factors bind alone or in clusters to the DNA and can thus activate or impede transcription. Many of the transcription factors can bind to several, different DNA sequence motifs with varying strength [1]. Recent studies suggest that not only strong interactions between transcription factors and the DNA influence gene expression, but that weak interactions significantly contribute to transcriptional regulation and are evolutionary conserved [2]. Quantitative models support the importance of weak interactions and show that correct recapitulation of transcriptional processes is only possible by including low-affinity transcription factor binding sites in their calculations [3]. Hence, in order to get a comprehensive picture of transcriptional regulation, it is essential to quantify the interaction of a broad range of transcription factors with all possible DNA sequences.

Recent developments in high-throughput techniques, for example the *in vivo* method chromatin immunoprecipitation combined with microarray analysis (ChIP-chip) [4], [5] or sequencing (ChIP-seq) [6] or *in vitro* techniques like protein binding microarrays (PBM) [7]–[10] have greatly increased our knowledge about various transcription factor binding sites. However, in most instances these techniques lack the ability to accurately quantify the protein-DNA interaction or require complicated algorithms and approximations to do so. Various methods exist to characterize the protein-DNA interactions by measuring thermodynamic and kinetic constants, for example electrophoretic mobility shift assay (EMSA) or surface plasmon resonance. Yet their common drawback is the low throughput that makes it nearly impossible to analyze a transcription factor against a whole genome. Two techniques have made huge advances in bridging the gap between measuring thermodynamic constants and high throughput, namely mechanically induced trapping of molecular interactions (MITOMI) [11] and high-throughput sequencing - fluorescent ligand interaction profiling (HiTS-FLIP) [12]. Both can determine dissociation constants of several transcription factors against thousands of DNA sequences (MITOMI) or of one protein against millions of DNA motifs (HiTS-FLIP), but require some approximations in order to calculate dissociation constants in a high-throughput format (MITOMI) or need a washing step that interferes with the analysis of transient interactions (HiTS-FLIP).

Importantly, due to the high concentration of DNA in a bacterial cell or eukaryotic nucleus, the dynamic equilibrium between unbound and bound activated transcription factors is shifted towards DNA-protein complexes. Hence, affinity described by the dissociation constant might not be the best measure to characterize the protein-DNA interaction inside a nucleus. The specificity defined as the ability of a transcription factor to discriminate between a regulatory sequence and the vast majority of non-regulating DNA might be a more suitable quantity. But quantification of the specificity in that sense means to determine the complete list of dissociation constants for all possible DNA sequences or a constant calculated from those dissociation constants [13]. Therefore, a method that determines the specificity in a single measurement is highly desirable considering the number of transcription factors and possible genomic sequences. Since the force required to break a bond increases with decreasing potential width, a more localized interaction between protein and DNA as it is expected for a sequence specific interaction will result in a higher unbinding force. Thus, a possibility for describing the specificity arises out of the binding strength between a protein and a DNA motif that is accessible in force-based measurements. Single-molecule force spectroscopy experiments allow the characterization of a protein-DNA bond in great detail [14]–[18] but are very time consuming and therefore not the appropriate tool to analyze the binding properties of a transcription factor against a whole genome.

The Molecular Force Assay (MFA) developed in our lab [19], [20] parallelizes single-molecule force experiments. It relies on the principle of comparing the interaction in question with a well-defined reference bond. We here describe a new application of the MFA to quantify binding strengths of several DNA-protein complexes directly and in parallel. This should contribute to a more conclusive and complete understanding of transcriptional regulation. In an adaptation of the original setup, we demonstrate in a proof-of principle experiment that we are able to determine the binding strength of a zinc finger protein against several DNA sequences in a single measurement.

Zinc finger motifs are one of the most abundant DNA binding domains in eukaryotic transcription factors [21]. The protein in our experiment Zif268/NRE is an artificial fusion protein of two zinc fingers of the Cys_2_-His_2_ class [22]. Zif268 is a transcription factor in mouse and a popular model system due to the existence of structural data of the protein-DNA complex [21], [23]. NRE is an engineered variant of Zif268 that binds specifically and with high affinity to a nuclear receptor element [24]. Our force-based design allows us to characterize the interaction of this six zinc finger protein with three DNA binding motifs, a high affinity sequence, a low affinity sequence and a no binding sequence, by a single value that can be directly correlated to the binding strength. Additionally, we show that we could gain further information about differences in the binding strength by varying the reference bond between a 20 base pair (bp) DNA sequence and a 40 bp DNA sequence. This demonstrates the possibility to convert the measured binding strength into intuitive units of DNA base pairs binding strength. Hence, this new variant of the MFA can quantify DNA-protein interaction and describe the binding strength in a simple picture by correlating it to the average binding strength of a certain number of DNA base pairs.

## Results and Discussion

The standard Molecular Force Assay (MFA) consists of two molecular bonds in series, a reference and a sample bond, clamped between two surfaces. The two surfaces are separated with a constant velocity so that a force builds up in the two molecular bonds until the weaker one ruptures. A fluorophore conjugated to the linker sequence between the two molecular complexes indicates the intact molecular bond. Hence, the ratio of the fluorescence intensity before and after the force loading of the molecular constructs is a measure of the strength of the sample bond in comparison to the reference bond. An alternative view of this assay is that the force greatly enhances the off rate of the bond under investigation and reduces the otherwise extremely long spontaneous dissociation times towards seconds [25]. As every molecular complex is tested against its own reference bond, the measurement is a single-molecule experiment that can be conducted in parallel with several thousand constructs. If oligonucleotide sequences are used for sample and reference complex, different binding sequences for ligands can be introduced in the sample bond so that a strengthening of the sample bond can be detected upon binding. Thus, the dissociation constant for ligands like polyamides [26] or proteins [27] was determined and an ATP-aptamer [28] as well as the interaction of the protein Dicer with double-stranded RNA [29] was characterized. Additionally, the reference bond can be varied in length and thus in the binding strength the sample bond is compared to. Hence, it was possible in former studies to quantify the increase of the sample bond strength upon ligand binding to the stability of 9.5 base pairs for a polyamide and to 27.7 base pairs for the protein EcoRI [30]. In a subsequent experiment integrated in a microfluidic setup, the binding of EcoRI to two sample bonds with different affinity was tested against four different reference bonds in a single measurement and the stabilization of the sample bonds was quantified in units of DNA base pairs. [31].

**Figure 1:**
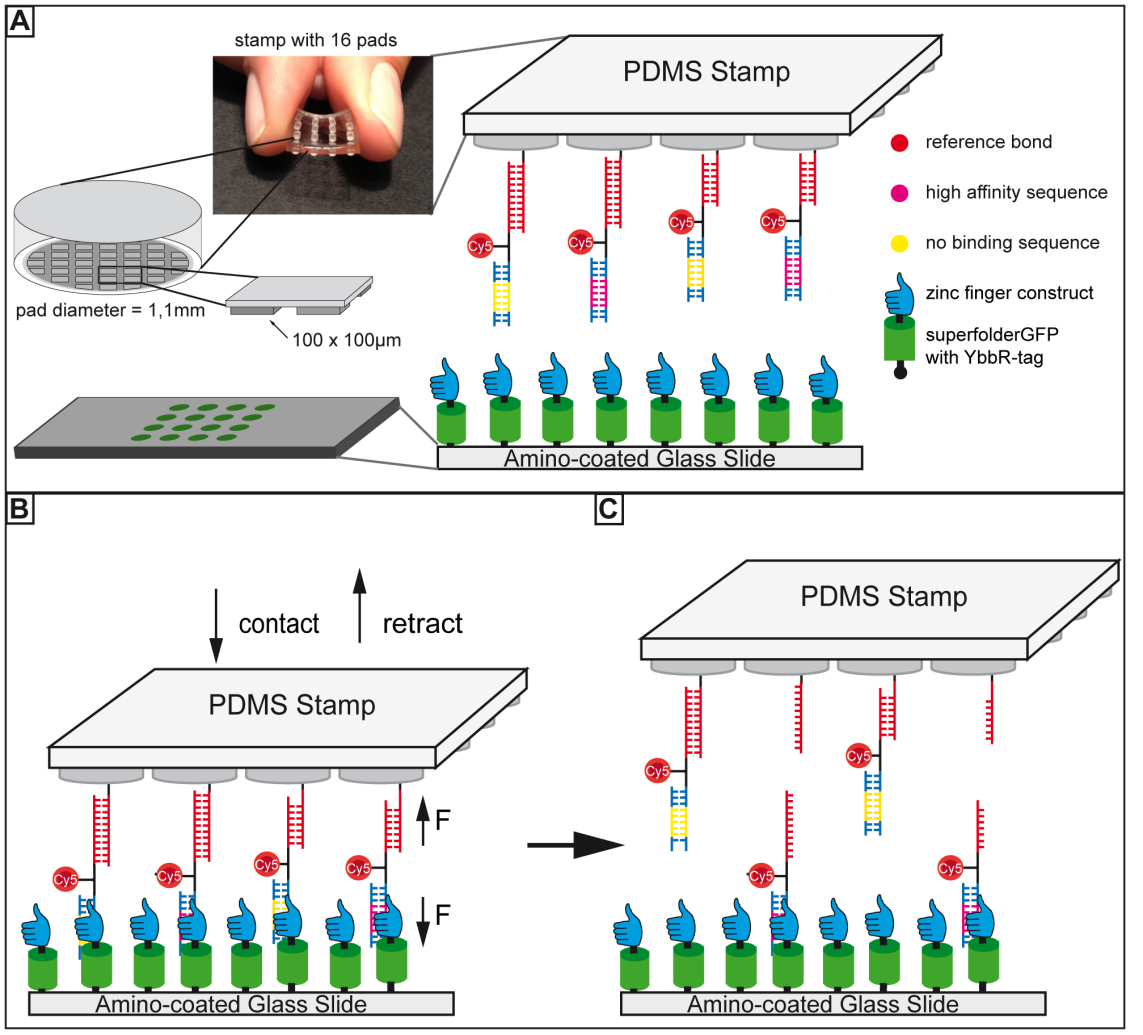
Description of the Molecular Force Assay (MFA). (A) The geometries of the PDMS stamp and the 4x4 pattern of protein spots on the glass slide are displayed. The zinc finger protein is covalently bound to an amino-coated glass slide functionalized with Coenzyme A via a ybbR-tag. A superfolderGFP acts as an additional spacer and helps to adjust the glass slide beneath the pads of the stamp. Different combinations of reference sequences and DNA binding motifs are attached to each pillar. (B) The PDMS stamp is carefully brought into contact with the glass slide and the DNA sample bonds are allowed to bind to the protein. Subsequently, the PDMS stamp is retracted with constant velocity so that a force builds up in the DNA-protein complexes and the reference bonds until the weaker construct ruptures. (C) After the force probe, the fluorescence signal on the glass slide is a measure for the number of intact protein-DNA bonds.

In the configuration of the MFA used in all former studies, the ligand-DNA interaction is not directly probed, but the ligand stabilizes the molecular bond and is thus detected. We here describe our new variant of the MFA that can probe the protein-DNA interaction directly and compare it to a reference bond. For this purpose, the fusion protein construct consisting of an N-terminal ybbR-tag [32] followed by a superfolderGFP [33] variant and the six zinc finger construct ZIF268/NRE [22] (details can be found in Supplement S1) is covalently attached via the ybbR-tag to a glass slide coated with Coenzyme A in a 4x4 pattern [34]. The two double-stranded DNA complexes in series are covalently attached to the 16 pillars of a soft PDMS surface with the upper one as reference bond and the lower one as sample bond (see Figure 1A). The DNA sequences in shear geometry are separated by a linker sequence to which a Cy5 fluorophore is conjugated. Due to the macrostructure of the PDMS stamp (see Figure 1A) a maximum of 16 combinations of different reference sequences as well as sample sequences can be tested within one experiment (Figure 1A). The PDMS surface is carefully brought into contact with the glass slide so that the sample sequence is able to bind to the protein on the glass slide (Figure 1B). This process is controlled via reflection interference contrast microscopy [35]. The GFP signal is used to place the protein spots below the stamp pillars functionalized with the different DNA sequences. After 10 minutes, the PDMS surface is retracted with constant velocity by a Piezo actuator. Thereby, a force is applied to the protein-sample complex as well as to the reference bond until the weaker one ruptures (Figure 1C). The fluorescence Cy5 signal on the glass slide is measured by an inverted epi-fluorescence microscope and indicates the number of intact protein-DNA complexes. Thus, the protein-DNA interaction is directly probed and compared to a well-characterized DNA double strand. In order to approximate the environment in a eukaryotic nucleus we designed our experiments as a competition assay and pre-incubated the zinc finger protein with low-molecular weight DNA from salmon sperm before the contact process. Details on the surface funtionalization, molecular constructs, contact and separation process as well as the fluorescence read-out are described in Supplement S1.

**Figure 2:**
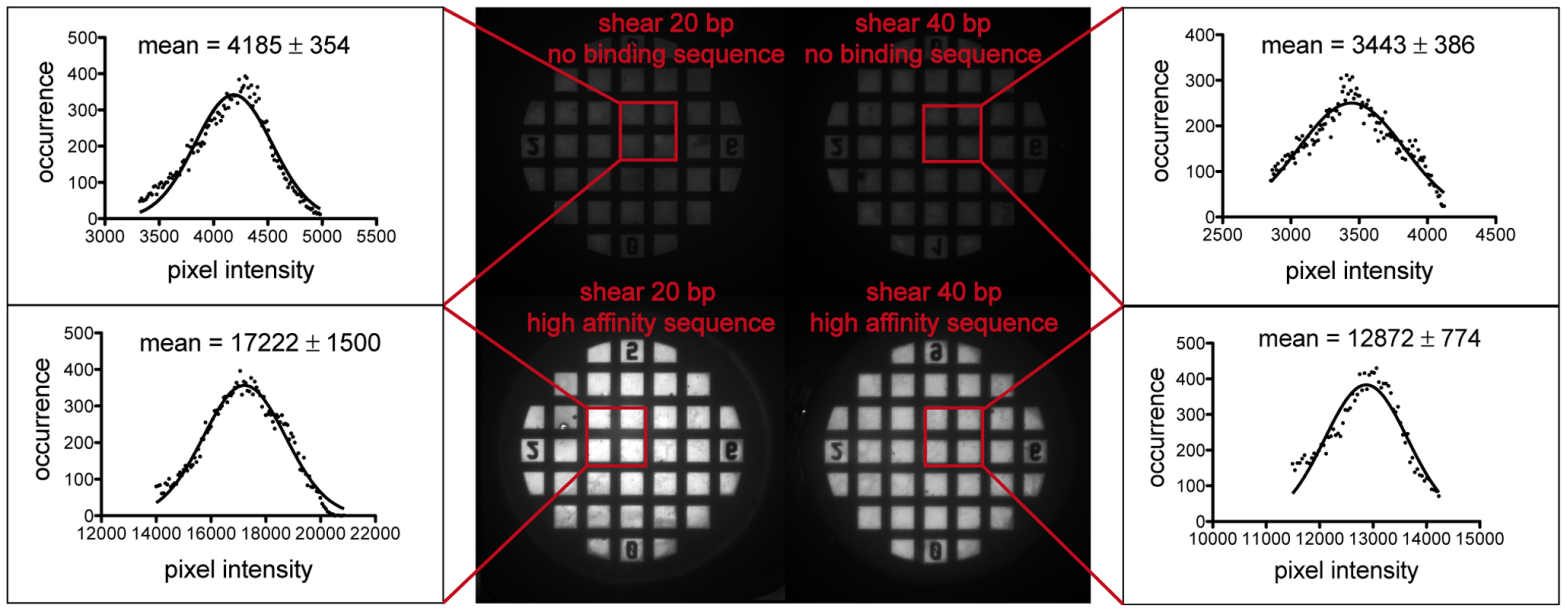
Transfer of Cy5-labeled DNA to the glass slide. After the contact and separation process, the fluorescence intensity of Cy5 on the glass slide is determined. Histograms of selected areas (without prior background subtraction) show a very modest signal slightly above the background signal (1000–2000 counts) for the DNA harboring the no binding sequences for the protein in question. DNA with a high affinity sequence did bind the protein in question and a transfer signal is clearly visible. The images are optimized in contrast to make the transfer of the no binding sequence as well as the difference in fluorescence signal between the no binding sequence and high affinity motif visible. A first assessment of the binding strength is possible by varying the reference bond. The weaker reference of 20 bp shows a higher fluorescence intensity of 17000 counts compared to the stronger reference of 40 bp with 13000 counts.

In a first test of our assay, we determined the binding of the zinc finger protein to a no binding sequence and a high affinity binding motif. The bond strength was compared to two reference sequences, a 20 bp double-stranded DNA and a 40 bp double-stranded DNA, both in shear geometry, by measuring the Cy5 fluorescence intensity of the transferred DNA after the contact and separation process. Figure 2 displays the results for all possible combinations of sample and reference bond. For the no binding sequence, only very little signal is measured. It hardly exceeds the background value of about 1000–2000 counts of pixel intensity so that false positives of unspecific interactions between the zinc finger protein with no binding sequences can be excluded in our assay. The high affinity sequence on the other hand clearly bound to the protein and the upper reference bond ruptured in most cases so that Cy5 labeled DNA was transferred to the glass slide. Additionally, a difference between the two reference bonds is evident. The weaker reference of 20 bp ruptured more often, yielding 17000 counts of transferred DNA on the slide. The stronger reference exceeds the binding strength of the protein-high affinity sequence interaction in more cases than the weaker reference, yielding distinctly less fluorescence signal of 13000 counts. These results of our first test confirm the specificity and feasibility of our approach for quantifying DNA-protein binding strength by means of the MFA and varying reference bonds.

**Figure 3:**
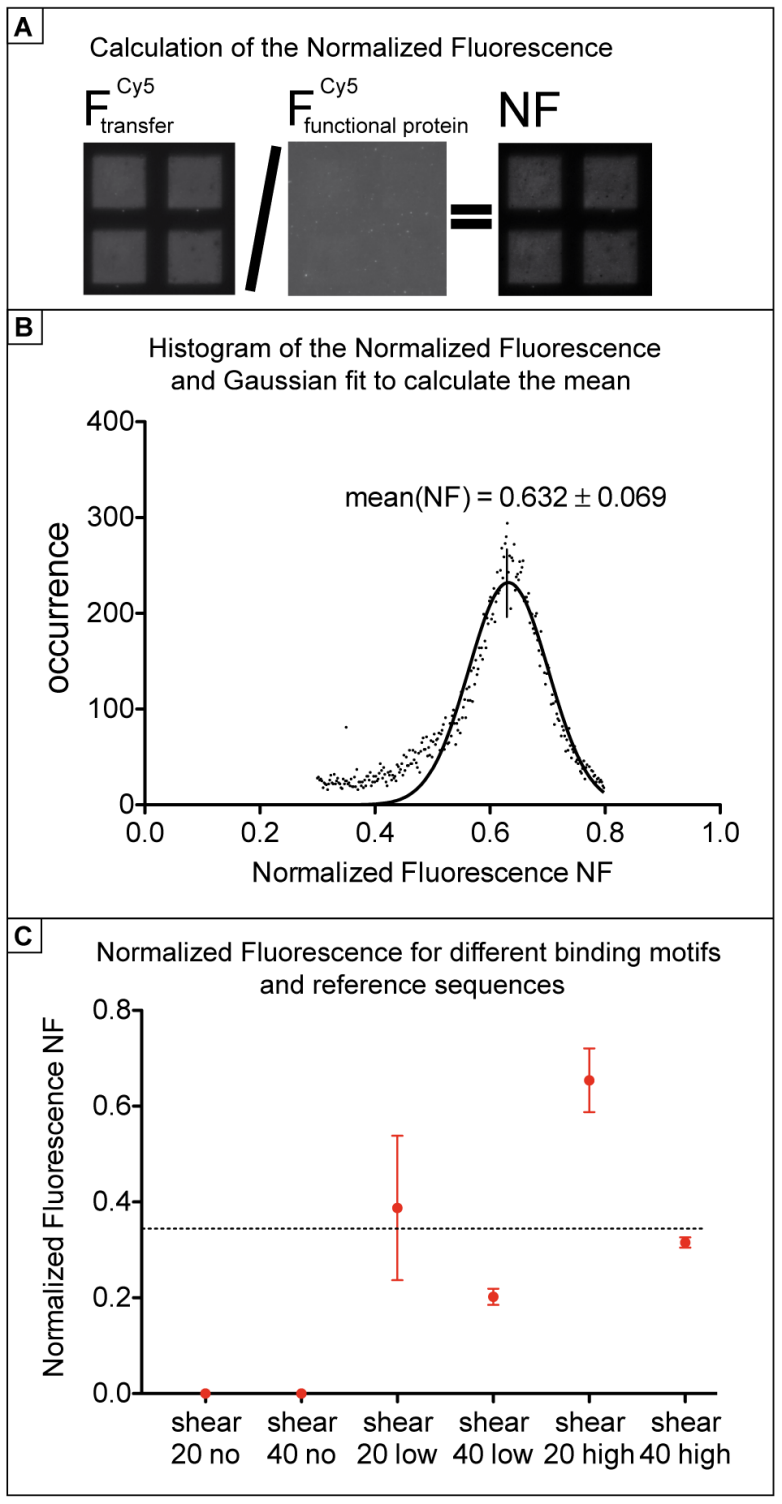
Quantification of the binding strength. (A) In order to quantify the binding strength, the flurorescence signal representing the DNA transfer has to be normalized to the number of available protein binding sites. For this purpose, a Cy5-labeled 40 bp DNA duplex harboring a high affinity binding motif is added subsequently to the force measurement in order to saturate all functional proteins. Following a washing protocol to remove all unbound DNA strands, the fluorescence intensity is measured a second time. After background subtraction, the fluorescence intensity of transferred DNA is divided by the signal corresponding to all functional proteins, yielding the Normalized Fluorescence NF. (B) Histograms of every pad on the PDMS stamp sum up the huge number of single-molecule experiments and are fitted by a Gaussian distribution in order to calculate an average NF and the standard deviation. Here, the histogram of the NF displayed in A is shown in detail. (C) One example measurement is displayed as a proof-of-principle. Details to the statistics are described in Supplement S1. The NF for the no binding sequences is too little to render fitting procedures possible. So we approximate the NF to be zero. Differences between low and high affinity binding motifs are very pronounced. A variation of the reference bond between 20 and 40 bp shear shows that the NF of the low affinity sequence against a 20 bp shear is about the same a the NF of the high affinity sequence against a 40 bp shear. This can be descriptively interpreted such that the difference in binding strength of the zinc finger protein with a low affinity sequence compared to a high affinity sequence corresponds to the stability of 20 bp DNA duplex.

In order to calculate a single, comparable number for the binding strength, environmental differences like the binding density of protein and oligonucleotide constructs on the surfaces have to be taken into account. In order to correct for differences in protein density on the glass slide, 0.5 µM of a Cy5 labeled 40 bp DNA duplex carrying a high affinity binding site for the protein in question is added subsequent to the force probe experiment to saturate all functional proteins bound to the surface. Calibration measurements confirmed a complete saturation after 30 min incubation time. After removing unbound fluorophores by a washing step, the fluorescence on the glass slide is determined again. It is a measure for the maximum number of functional proteins on the slide. Since the binding density of the DNA complexes on the PDMS always exceeds the number of functional proteins on the glass slide, further corrections are not necessary. The ratio of fluorescence signal on the glass slide directly after the rupture event F_transfer_ to the maximal number of functional proteins F_intact protein_ is defined as the Normalized Fluorescence, NF. The NF is calculated by dividing the pictures after background subtraction pixel-by-pixel (see Figure 3A), which cancels out inhomogeneities and renders this method robust. Histograms of the NF picture are generated and fitted by a Gaussian to yield the NF mean and standard deviation (Figure 3B). Thus, every mean value of the NF is the result of several million tested molecular constructs (more details about the statistics can be found in Supplement S1). This number can be interpreted as the binding strength of the protein-DNA interaction in comparison to a certain reference bond. A variation of the reference bond will result in a different NF and refines the information of the DNA-protein interaction. We tested our zinc finger protein against three DNA double strands incorporating either a high affinity sequence, a low affinity sequence or a no binding sequence against two reference bonds, a 20 bp and a 40 bp DNA double strand and analyzed the data in the way just described (the exact sequences are shown in Figure S1). The result of one example experiment is depicted in Figure 3C. Due to the low DNA transfer for the no binding sequence, a calculation of the NF was not possible, so we set these values to zero. Differences are clearly visible for the NF values for the low and high affinity sequences as well as for the variations of the reference bond. As expected, we measured the highest value of 0.65±0.07 for the high affinity sequence against the 20 bp reference bond compared to 0.39±0.15 for the low affinity sequence against the same reference bond. The stronger reference bond lowers the values to 0.32±0.01 and 0.20±0.02 for high and low affinity DNA motifs, respectively. For both DNA binding motifs, the mean NF is reduced by half if the number of reference base pairs is doubled: 0.65 (20 bp) to 0.32 (40 bp) for the high affinity motif and 0.39 (20 bp) to 0.20 (40 bp). Hence, a linear relationship between the number of reference base pair and the mean NF can be assumed in this range of reference bond length. This result does not mean that the strength of the protein-DNA bond is altered by different reference bonds. The comparison of the protein-DNA bond with different reference bonds yields different NF values that draw a more detailed picture of the protein-DNA interaction and enables to adjust the setup to the biological problem. A linear relationship between the NF and number of base pairs in the reference duplex makes it possible to adjust the reference duplexes until the NF yields a value of 0.5 so that the reference duplex of a certain number of base pairs has the same stability as the protein-DNA bond. Thus, the protein-DNA bond strength can be directly quantified with the stability of a certain number of base pairs. In our proof-of principle experiment, we compare the stability of a protein-DNA interaction with varying affinities to the stability of two DNA duplexes of different lengths. Interestingly, the NF values for the low affinity sequence against the 20 bp reference bond, 0.39, and for the high affinity sequence against the 40 bp reference bond, 0.32, are equal within errors (see Figure 3C). This allows the interpretation of a difference in binding strength of the zinc finger protein with these two DNA motifs that corresponds to the average binding strength of a 20 bp DNA double strand. Thus, we demonstrated that the specificity of DNA-protein interactions can be quantified via the binding strength in a force-based assay in a single measurement. Further, we can characterize the binding strength in a simple picture by correlating it to the average binding strength of a certain number of DNA base pairs.

## Conclusion

We described a new variant of the MFA that allows to directly detect the binding strength of protein-DNA interactions. This force-based format can test several DNA sequences against a protein in parallel with good statistics and can characterize the binding strength descriptively by correlating it to the average binding strength of a certain number of DNA base pairs. As a proof-of-principle, we could quantify the interactions of a zinc finger protein with three DNA sequences and compare them against two reference bonds. The resolution of the assay depends on the biological problem and the strength of the reference duplex. It was already demonstrated that the MFA can detect a single nucleotide polymorphism in a 20 base pair DNA duplex [19]. Shorter reference duplexes or a reference duplex in zipper geometry can discriminate between very small differences in the strength of the protein-DNA complexes invoked for example by a single base pair variation in the DNA target sequence. Further experiments will identify the capabilities and limitations of the assay for different DNA-protein complexes. For a complete characterization of a protein's binding specificity and affinity, it is necessary to probe the interactions with DNA sequences representative of a whole genome. This is, in principle, feasible with our force-based design. We have already shown that much smaller geometries for the DNA spots are sufficient to calculate the NF [27] and the fabrication of DNA microarrays is a standard procedure. Furthermore, our lab succeeded in integrating the MFA in a microfluidic chip [31]. The utilized surface chemistry also allows for the measurement of several proteins in a single experiment. Thus, our force-based assay can quantify protein-DNA interactions in a parallel format. It has the potential, with further developments in miniaturization and parallelization, to improve our understanding of transcriptional regulation.

## Supporting Information

Figure S1 DNA sequences.
(TIF)

Supplement S1 Materials and Methods.
(DOC)

## References

[1] Badis G, Berger MF, Philippakis AA, Talukder S, Gehrke AR, et al (2009) Diversity and complexity in DNA recognition by transcription factors. Science 324: 1720–1723. 19443739 10.1126/science.1162327 PMC2905877

[2] Tanay A (2006) Extensive low-affinity transcriptional interactions in the yeast genome. Genome Res 16: 962–972. 16809671 10.1101/gr.5113606 PMC1524868

[3] Segal E, Raveh-Sadka T, Schroeder M, Unnerstall U, Gaul U (2008) Predicting expression patterns from regulatory sequence in Drosophila segmentation. Nature 451: 535–540. 18172436 10.1038/nature06496

[4] Ren B, Robert F, Wyrick JJ, Aparicio O, Jennings EG, et al (2000) Genome-wide location and function of DNA binding proteins. Science 290: 2306–2309. 11125145 10.1126/science.290.5500.2306

[5] Iyer VR, Horak CE, Scafe CS, Botstein D, Snyder M, et al (2001) Genomic binding sites of the yeast cell-cycle transcription factors SBF and MBF. Nature 409: 533–538. 11206552 10.1038/35054095

[6] Park PJ (2009) ChIP-seq: advantages and challenges of a maturing technology. Nat Rev Genet 10: 669–680. 19736561 10.1038/nrg2641 PMC3191340

[7] Bulyk ML, Huang X, Choo Y, Church GM (2001) Exploring the DNA-binding specificities of zinc fingers with DNA microarrays. Proc Natl Acad Sci U S A 98: 7158–7163. 11404456 10.1073/pnas.111163698 PMC34639

[8] Mukherjee S, Berger MF, Jona G, Wang XS, Muzzey D, et al (2004) Rapid analysis of the DNA-binding specificities of transcription factors with DNA microarrays. Nat Genet 36: 1331–1339. 15543148 10.1038/ng1473 PMC2692596

[9] Berger MF, Philippakis AA, Qureshi AM, He FS, Estep PW, et al (2006) Compact, universal DNA microarrays to comprehensively determine transcription-factor binding site specificities. Nat Biotechnol 24: 1429–1435. 16998473 10.1038/nbt1246 PMC4419707

[10] Berger MF, Bulyk ML (2009) Universal protein-binding microarrays for the comprehensive characterization of the DNA-binding specificities of transcription factors. Nat Protoc 4: 393–411. 19265799 10.1038/nprot.2008.195 PMC2908410

[11] Fordyce PM, Gerber D, Tran D, Zheng J, Li H, et al (2010) De novo identification and biophysical characterization of transcription-factor binding sites with microfluidic affinity analysis. Nat Biotechnol 28: 970–975. 20802496 10.1038/nbt.1675 PMC2937095

[12] Nutiu R, Friedman RC, Luo S, Khrebtukova I, Silva D, et al (2011) Direct measurement of DNA affinity landscapes on a high-throughput sequencing instrument. Nat Biotechnol 29: 659–664. 21706015 10.1038/nbt.1882 PMC3134637

[13] Stormo GD, Zhao Y (2010) Determining the specificity of protein-DNA interactions. Nat Rev Genet 11: 751–760. 20877328 10.1038/nrg2845

[14] Koch SJ, Shundrovsky A, Jantzen BC, Wang MD (2002) Probing protein-DNA Interactions by Unzipping a Single DNA Double Helix. Biophysical Journal 83: 1098–1105. 12124289 10.1016/S0006-3495(02)75233-8 PMC1302211

[15] Bartels FW, Baumgarth B, Anselmetti D, Ros R, Becker A (2003) Specific binding of the regulatory protein ExpG to promoter regions of the galactoglucan biosynthesis gene cluster of Sinorhizobium meliloti - a combined molecular biology and force spectroscopy investigation. Journal of Structural Biology 143: 145–152. 12972351 10.1016/s1047-8477(03)00127-8

[16] Kuhner F, Costa LT, Bisch PM, Thalhammer S, Heckl WM, et al (2004) LexA-DNA bond strength by single molecule force spectroscopy. Biophys J 87: 2683–2690. 15454462 10.1529/biophysj.104.048868 PMC1304687

[17] Bizzarri AR, Cannistraro S (2010) The application of atomic force spectroscopy to the study of biological complexes undergoing a biorecognition process. Chem Soc Rev 39: 734–749. 20111790 10.1039/b811426a

[18] Moy VT, Florin EL, Gaub HE (1994) Intermolecular forces and energies between ligands and receptors. Science 266: 257–259. 7939660 10.1126/science.7939660

[19] Albrecht C, Blank K, Lalic-Multhaler M, Hirler S, Mai T, et al (2003) DNA: a programmable force sensor. Science 301: 367–370. 12869761 10.1126/science.1084713

[20] Blank K, Mai T, Gilbert I, Schiffmann S, Rankl J, et al (2003) A force-based protein biochip. Proc Natl Acad Sci U S A 100: 11356–11360. 12975526 10.1073/pnas.1934928100 PMC208761

[21] Pavletich NP, Pabo CO (1991) Zinc Finger-DNA Recognition: Crystal Structure of a Zif268-DNA Complex at 2.1 A. Science. 252: 809–817. 10.1126/science.2028256 2028256

[22] Kim J-S, Pabo CO (1998) Getting a handhold on DNA: Design of poly-zinc finger proteins with femtomolar dissociation constant. Proc Natl Acad Sci U S A 95: 2812–2817. 9501172 10.1073/pnas.95.6.2812 PMC19651

[23] Elrod-Erickson M, Rould MA, Nekludova L, Pabo CO (1996) Zif268 protein-DNA complex refined at 1.6 A: a model system for understanding zinc finger-DNA interactions. Structure 4: 1171–1180. 8939742 10.1016/s0969-2126(96)00125-6

[24] Greisman HA, Pabo CO (1997) A general strategy for selecting high-affinity zinc finger proteins for diverse DNA target sites. Science 275: 657–661. 9005850 10.1126/science.275.5300.657

[25] Carrion-Vazquez M, Oberhauser AF, Fowler SB, Marszalek PE, Broedel SE, et al (1999) Mechanical and chemical unfolding of a single protein: a comparison. Proc Natl Acad Sci U S A 96: 3694–3699. 10097099 10.1073/pnas.96.7.3694 PMC22356

[26] Ho D, Dose C, Albrecht CH, Severin P, Falter K, et al (2009) Quantitative detection of small molecule/DNA complexes employing a force-based and label-free DNA-microarray. Biophys J 96: 4661–4671. 19486688 10.1016/j.bpj.2009.02.059 PMC2711479

[27] Severin PM, Ho D, Gaub HE (2011) A high throughput molecular force assay for protein-DNA interactions. Lab Chip 11: 856–862. 21221429 10.1039/c0lc00302f

[28] Ho D, Falter K, Severin P, Gaub HE (2009) DNA as a force sensor in an aptamer-based biochip for adenosine. Anal Chem 81: 3159–3164. 19364143 10.1021/ac802766j

[29] Limmer K, Aschenbrenner D, Gaub HE (2013) Sequence-specific inhibition of Dicer measured with a force-based microarray for RNA ligands. Nucleic Acids Res 41: e69. 23303774 10.1093/nar/gks1455 PMC3616731

[30] Severin PM, Gaub HE (2012) DNA-Protein Binding Force Chip. Small. 10.1002/smll.201201088 22887737

[31] Otten M, Wolf P, Gaub HE (2013) Protein-DNA force assay in a microfluidic format. Lab Chip 13: 4198–4204. 23986395 10.1039/c3lc50830g

[32] Yin J, Lin AJ, Golan DE, Walsh CT (2006) Site-specific protein labeling by Sfp phosphopantetheinyl transferase. Nat Protoc 1: 280–285. 17406245 10.1038/nprot.2006.43

[33] Pedelacq JD, Cabantous S, Tran T, Terwilliger TC, Waldo GS (2006) Engineering and characterization of a superfolder green fluorescent protein. Nat Biotechnol 24: 79–88. 16369541 10.1038/nbt1172

[34] Wong LS, Thirlway J, Micklefield J (2008) Direct site-selective covalent protein immobilization catalyzed by a phosphopantetheinyl transferase. J Am Chem Soc 130: 12456–12464. 18722432 10.1021/ja8030278

[35] Wiegand G, Neumaier KR, Sackmann E (1998) Microinterferometry: three-dimensional reconstruction of surface microtopography for thin-film and wetting studies by interference contrast microscopy (RICM). Appl Opt 37: 6892–6905. 18301506 10.1364/ao.37.006892

