# Supplementary figures and images for: A Force-Based, Parallel Assay for the Quantification of Protein-DNA Interactions
Source: PLoS One. 2014 Feb 27;9(2):e89626. doi: 10.1371/journal.pone.0089626 (PMC3937344; doi:10.1371/journal.pone.0089626)

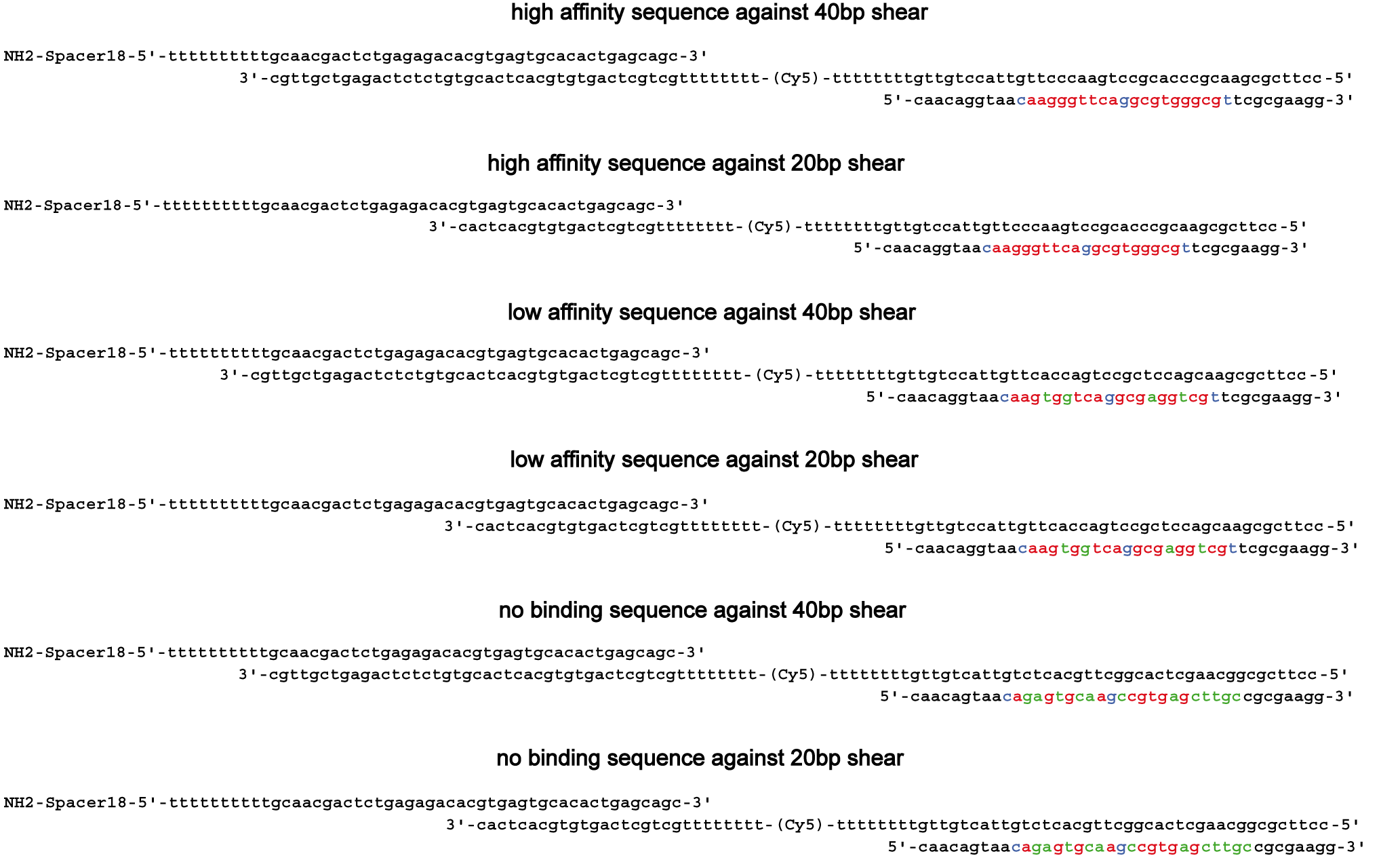

Supplement: Figure S1 — DNA sequences. (TIF) [file pone.0089626.s001.tif]
