# Supplementary material for: A Force-Based, Parallel Assay for the Quantification of Protein-DNA Interactions
Source: PLoS One. 2014 Feb 27;9(2):e89626. doi: 10.1371/journal.pone.0089626 (PMC3937344; doi:10.1371/journal.pone.0089626)
Supplement: Supplement S1 — Materials and Methods. (DOC) [file pone.0089626.s002.doc]

**Supplement**

**Materials and Methods**

Oligonucleotide constructs

The molecular complexes consist of three strands that are successively hybridized in shear geometry prior to usage. The uppermost strand which is covalently bound to the PDMS stamp is modified with an amino-group. Spacer18, a hexaethylene glycol chain of 18 atoms length, acts as an additional spacer between the amino-group and the oligonucleotides in order to avoid surface effects. Furthermore, poly-T stretches link the double-stranded sequences to the surfaces and each other. The cyanine dye Cy5 is attached by an N-Hydroxysuccinimide ester to the middle strand between the two duplexes.The reference bond is varied in length between 20 and 40 basepairs in order to test the protein-DNA bond against different strengths. The sample bond varies in its sequence in order to analyze the binding behavior of the protein against a high affinity DNA, 5’-caacaggtaac**aagggttca**g**gcgtgggcg**ttcgcgaagg-3', a low affinity DNA, 5'-caacaggtaac**aag*t*g*g*tca**g**gcg*a*gg*t*cg**ttcgcgaagg-3', and a no binding sequence, 5'-caacagtaacagagtgcaagccgtgagcttgccgcgaagg-3'. The complete DNA constructs are dispalyed in Figure S1. All oligonulceotide constructs, including modified ones, were obtained from biomers.net GmbH, Germany.

Protein construct

A fusion protein construct consisting of an N-terminal ybbR-tag (DSLEFIASKLA) followed by a superfolderGFP variant and the six zinc finger protein construct Zif268/NRE (with an RQKDGERP linker sequence between the Zif268 and NRE moieties) was cloned into pGEX6P2 between BamHI and XhoI sites similar to . All construct fragments were amplified from synthetic templates (Mr.Gene or Geneart, Lifetechnologies, UK). The resulting fusion protein (ybbR-sfGFP-Zif268/NRE) harbored a GST-tag and was expressed in *E.coli* BL21 DE3 Codon Plus cells (Agilent Technologies, USA). One liter of SB medium was inoculated with 10ml of an overnight culture and grown at 37°C. When an OD_600_ of 0.7 had been reached, over night expression at 18°C was induced by adding 0.25mM IPTG. Cells were lyzed in 50mM TRIS-HCl (pH 7.5, 300 mM NaCl, 2mM DTT, 5% Glycerol, 10µM ZnCl_2_) by a French pressure cell press. The ybbR-GFP-zinc finger construct was obtained in the soluble fraction and purified by Glutathione affinity chromatography on GSTrap columns (GE Healthcare, Germany) according to standard procedures. After over night treatment with PreScission protease the GST-tag was removed and the protein further purified by Heparin cation exchange chromatography (HiTrap Heparin, GE Healthcare, Germany). Following preparative size exclusion chromatography on a HiLoad 16/60 Superdex 75 column (GE Healthcare, Germany) in 50mM TRIS-HCl (pH7.5, 150mM NaCl, 2mM DTT, 10µM ZnCl_2_, 5% Glycerol) the protein construct was concentrated to 10µM by ultrafiltration in Amicon Ultra centrifugal filter units (Merck Millipore, USA) and stored at -80°C until further usage.

Stamp preparation

Micro-and macrostructured poly(dimethylsiloxane) (PDMS) stamps were fabricated by casting 1:10 crosslinker/base (Sylgard, Dow Corning, MI, USA) into a custom-made Pyrex/silicon wafer (HSG-IMIT, Germany) according to standard procedures . The resulting PDMS stamps feature pillars of 1mm diameter and height with a spacing of 3mm in a square pattern on a 3mm thick basis and are cut in pieces of 4x4 pillars. The flat surface of the pillars is microstructured with 100µm x 100µm pads separated by 41µm wide and 5µm deep rectangular trenches enabling the drainage of liquid during the contact and separation process (Figure 1A). For the surface functionalization, the cleaned stamp surface was first activated in 12.5% HCl overnight and derivatized with (3-glycidoxypropyl)-trimethoxysilane (ABCR, Germany) in order to generate epoxide groups. After 30 minutes at 80°C in an Argon atmosphere, the functionalized stamp was allowed to cool down to room temperature. The amino-modified DNA strand, dissolved in water, was diluted 1:10 in a sodium borate-buffer (50mM H_3_BO_3_, 50mM Na_2_B_4_O_7_•10 H_2_O pH=9.0; Carl Roth GmbH & Co. KG, Germany) to a concentration of 10µM and 1.5µl was transferred to every pillar on the stamp. Overnight incubation of the stamp under humid conditions allowed the amino-groups to react with the epoxide-groups. Oligonucleotides that did not bind to the stamp were washed off the next day in an aqueous solution of 0.01% SDS (sodium dodecyl sulphate; Sigma-Aldrich GmbH, Germany). The other two DNA strands were pre-incubated in 5x SSC buffer (saline sodium citrate; 750mM sodium chloride, 75mM trisodium citrate; Sigma-Aldrich GmbH, Germany) in a concentration of 0.2µM. 1.5µl was transferred to every pillar of the stamp. After a minimum of 60 minutes incubation time the functionalized stamp was washed with 0.05% Tween 20 (VWR Scientific GmbH, Germany) in 1x SSC and gently dried with N_2_ gas.

Slide preparation

Conventional glass slides for microscopy were aminosilanized in our lab: After thorough cleaning by sonication in 50% (v/v) 2-propanol in ddH20 for 15 min and oxidation in a solution of 50% (v/v) hydrogen peroxide (30%) and sulfuric acid for 30 min, they were washed with ddH2O and dried in a nitrogen stream. For the silanization, the glass slides were soaked for 1 h in a solution of 90% (v/v) ethanol, 8% ddH2O and 2% 3-aminopropyldimethylepoxysilane (ABCR, Germany). Subsequently they were washed twice in 2-propanol and ddH2O and dried at 80 °C for 40 min. They can be stored for several weeks in an Argon atmosphere at room temperature.

For further functionalisation, the amino-silanized glass slide was first deprotonated in a sodium borate buffer (50mM H_3_BO_3_, 50mM Na_2_B_4_O_7_•10 H_2_O pH=8.5; Carl Roth GmbH & Co. KG, Germany) for 30 minutes, then a heterobifunctional PEG crosslinker with N-hydroxy succinimide and maleimide groups (MW 5000, Rapp Polymere, Germany) was applied for 1 h at 30mM. The slide was thoroughly washed with ddH20 and gently dried with N2, before it was incubated another hour with Coenzyme A (Merck Millipore, USA) dissolved in coupling buffer (50mM NaHPO_4_, 50mM NaCl, 10mM EDTA at pH=7.2). Again the slide was washed with ddH2O and gently dried with N_2_. At this stage, the slide can be stored up to several days.

The Zif268/NRE protein aliquot at a concentration of 10µM is spun down in a table top centrifuge to remove agglomerates and the supernatant was diluted in a 50mM TRIS-HCl buffer (pH=7.5, 150mM NaCl, 10mM MgCl_2_, 10µM ZnCl_2_, 2mM DTT) to a final concentration of 2.5µM. Furthermore, low molecular weight DNA from salmon sperm (Sigma-Aldrich GmbH, Germany) was added in a concentration of 1g/ml. After a short incubation time of 15 minutes, 1,5 µl of Phosphopantetheinyltransferase Sfp was added to the sample and 2µl droplets of the mix were transferred to the functionalized glass slide in a 4x4 pattern. Sfp reacted the Coenzyme A on the glass slide to the ybbR-tag of the protein in humid atmosphere at room temperature during three hours incubation time. A PMMA mask with a well for the 4x4 pattern of spotted protein sample was fixed to the glass slide with a silicone lip seal. The mask prevented samples from drying out during following washing procedures and the MFA experiment. All protein that did not bind to the surface was washed off by 25ml 50mM TRIS-HCl buffer (pH=7.5, 150mM NaCl, 10µM ZnCl_2_), 25ml 100mM TRIS-HCl buffer (pH=7.5, 300mM NaCl, 10µM ZnCl_2_) and again 25ml 50mM TRIS-HCl buffer (pH=7.5, 150mM NaCl, 10µM ZnCl_2_). The last buffer was also used for the MFA experiments. After the washing procedure, the samples were measured within 3 hours.

Contact process and fluorescence read-out

The functionalized stamp adhered upside-down to the glass block glued to a closed-loop piezoelectric actuator (PZ 400, Piezo Systems Jena, Germany) and a DC motorized translation stage (Physik Instrumente GmbH, Germany). The slide with the oligonucleotide constructs was fixed beneath the stamp on a stainless steel stage with permanent magnets. The fluorescence signal of the superfolderGFP fused between the ybbR-Tag and the zinc finger protein was used to place every protein spot beneath the right stamp pillar. The whole contact device is mounted on an inverted microscope (Axio Observer Z1, Carl Zeiss MicroImaging GmbH, Germany) with an xy-DC motorized high-accuracy translation stage (Physik Instrumente GmbH, Germany). Contact was made by means of the piezo and care was taken that each individual pillar is not compressed more than 3µm. The planar adjustment of stamp and slide as well as the contact process were controlled by reflection interference contrast microscopy . In order to let the protein bind to the DNA sample sequence on the PDMS stamp, the contact between stamp and slide was maintained for 10 minutes. The piezo retracted the stamp with a velocity of 1µm/s in all experiments. A force buildt up in the molecular complexes until the weaker bond, either the protein-DNA complex or the reference bond, broke with higher probability. A Cy5 fluorophore conjugated to the linker sequence between the two DNA double strands indicated the intact bond. Hence, the Cy5 fluorescence intensity F_transfer_ on the glass slide was measured with a CCD camera (ANDOR iXon, Andor, Northern Ireland) after the contact and separation process. In order to normalize the signal of the intact protein-DNA complexes to the protein density on the glass slide, the sample was subsequently incubated with a 40 bp double-stranded DNA sequence containing the high affinity binding site and labeled with a Cy5 fluorophore in a concentration of 0.5µM for 30 minutes. Unbound dsDNA was removed by the following washing procedure: 25ml 50mM TRIS-HCl buffer (pH=7.5, 150mM NaCl, 10µM ZnCl_2_), 25ml 100mM TRIS-HCl buffer (pH=7.5, 300mM NaCl, 10µM ZnCl_2_) and again 25ml 50mM TRIS-HCl buffer (pH=7.5, 150mM NaCl, 10µM ZnCl_2_). The Cy5 fluorescence intensity was measured again and gives the number of possible protein binding sites. Since the binding density of the DNA complexes on the PDMS always exceeds the number of functional proteins on the glass slide, further corrections are not necessary. The ratio of fluorescence signal on the glass slide directly after the rupture event F_transfer_ to the maximal number of functional proteins F_intact protein_ is defined as the Normalized Fluorescence, NF. The NF is calculated by dividing the pictures after background subtraction pixel-by-pixel by a custom-built software written in Labview. Histograms of the NF picture are generated and fitted by a Gaussian to yield the NF mean and standard deviation.

Statistics

In every experiment, every pillar of the PDMS stamp can be functionalized with a different combination of reference and sample complex. In our proof-of-principle measurements we usually bind the same combination of sample and reference bond to at least two pillars for better statistics. The contact area of a pillar is (100x100 µm^2^ x 25)= 25x10^4^ µm^2^. From the fluorescence signal of the functional protein we can estimate a lower bound for the density of functional protein on the glass slide of 10^3^ per µm^2^. Thus, every pillar tests around 25x10^7^ molecular complexes and the NF is the mean of 25x10^7^ tested molecular complexes. In order to demonstrate the validity of our approach to quantify the specificity of the protein-DNA interaction in a single measurement with good statistics, we show the result of one example measurement. Every data point is the average of two mean NF values. All NF values in this measurement are very close except the one for the low affinity binding motif against the 20bp reference. Other experiments yielded results in good agreement with the displayed experiment.

**References**

1. Yin J, Lin AJ, Golan DE, Walsh CT (2006) Site-specific protein labeling by Sfp phosphopantetheinyl

transferase. Nat Protoc 1: 280-285.

2. Pedelacq JD, Cabantous S, Tran T, Terwilliger TC, Waldo GS (2006) Engineering and

characterization of a superfolder green fluorescent protein. Nat Biotechnol 24: 79-88.

3. Kim J-S, Pabo CO (1998) Getting a handhold on DNA: Design of poly-zinc finger proteins with

femtomolar dissociation constant. Proc Natl Acad Sci 95: 2812-2817.

4. Strackharn M, Pippig DA, Meyer P, Stahl SW, Gaub HE (2012) Nanoscale arrangement of proteins

by single-molecule cut-and-paste. J Am Chem Soc 134: 15193-15196.

5. Xia Y, Whitesides GM (1998) Soft Lithography. Annu Rev Mater Sci 28: 153-184.

6. Wiegand G, Neumaier KR, Sackmann E (1998) Microinterferometry: three-dimensional

reconstruction of surface microtopography for thin-film and wetting studies by interference contrast

microscopy (RICM). Appl Opt 37: 6892-6905.

**Figure S1**

**DNA sequences**. The molecular constructs with all modifications are displayed. The reference bond comprises the same sequence for all six constructs, but differs in the length of the middle strand. The ZIF268/NRE high affinity sequence is shown in red. The mutations for the low affinity sequence and the no binding sequence are colored green.
